# Anorexia Nervosa during Adolescence Is Associated with Decreased Gray Matter Volume in the Inferior Frontal Gyrus

**DOI:** 10.1371/journal.pone.0128548

**Published:** 2015-06-11

**Authors:** Takashi X. Fujisawa, Chiho Yatsuga, Hiroyo Mabe, Eiji Yamada, Masato Masuda, Akemi Tomoda

**Affiliations:** 1 Research Center for Child Mental Development, University of Fukui, Fukui, Japan; 2 Department of Child Development, United Graduate School of Child Development, Osaka University, Kanazawa University, Hamamatsu University School of Medicine, Chiba University and University of Fukui, Fukui, Japan; 3 Department of Child Development, Faculty of Life Sciences, Kumamoto University, Kumamoto, Japan; Chiba University Center for Forensic Mental Health, JAPAN

## Abstract

Anorexia nervosa (AN) is an eating disorder characterized by the relentless pursuit to lose weight, mostly through self-starvation, and a distorted body image. AN tends to begin during adolescence among women. However, the underlying neural mechanisms related to AN remain unclear. Using voxel-based morphometry based on magnetic resonance imaging scans, we investigated whether the presence of AN was associated with discernible changes in brain morphology. Participants were 20 un-medicated, right-handed patients with early-onset AN and 14 healthy control subjects. Group differences in gray matter volume (GMV) were assessed using high-resolution, T1-weighted, volumetric magnetic resonance imaging datasets (3T Trio scanner; Siemens AG) and analyzed after controlling for age and total GMV, which was decreased in the bilateral inferior frontal gyrus (IFG) (left IFG: FWE corrected, *p* < 0.05; right IFG: uncorrected, *p* < 0.05) of patients with AN. The GMV in the bilateral IFG correlated significantly with current age (left IFG: *r* = -.481, *p <* .05; right IFG: *r* = -.601, *p <* .01) and was limited to the AN group. We speculate that decreased IFG volume might lead to deficits in executive functioning or inhibitory control within neural reward systems. Precocious or unbalanced neurological trimming within this particular region might be an important factor for the pathogenesis of AN onset.

## Introduction

Anorexia nervosa (AN) is an eating disorder that generally begins during late childhood or adolescence among females. AN’s core psychopathology is characterized by the relentless pursuit of weight loss, mostly through self-starvation, and a distorted body image [1]. Based on recent epidemiology research, the lifetime prevalence of AN ranges from 0.5% to 2% [2], with a peak age at onset of 13 to 18 years [3]. Additionally, AN has a tendency toward chronification and a mortality rate of 7% [4]. Currently, the exact cause of AN is still unknown; interactions between genetic and biological diathesis, environmental and sociocultural influences, and psychological traits have been assumed [5]. Evidence for the heritability of eating disorders has increased, with AN showing rates between 33% and 84% in certain twin studies [2]. Based on this evidence, neurobiological factors are now being studied in AN; yet the neural mechanisms underlying the cause and persistence of the disorder remain poorly understood [6–7].

Several studies using brain imaging techniques have identified structural and functional abnormalities in cerebral regions among patients with AN. Previous structural imaging studies have consistently observed reduced gray matter (GM) and white matter (WM) volumes and increased cerebrospinal fluid among AN patients [8–12]. Other studies have reported an association between brain volume loss and AN severity [10,13–14] and persistence of GM reduction [15–16]. In recent years, the automated voxel-based morphometry (VBM) technique has been used to measure brain volume differences, which allows for a statistical, voxel-by-voxel assessment of differences across the whole brain. VBM enables the avoidance of operator-dependent manual tracing, a time-consuming technique susceptible to human error. Additionally, VBM allows for statistical estimations between clinical samples and controls on a whole or local brain level [17]. However, while the VBM approach is powerful for examining specific structural regions potentially related to AN pathology, this technique has shown variable results among AN samples. One recent meta-analysis of nine VBM studies revealed that AN patients not only have global reductions in GM, WM, and increased CSF but also show regional decreases in reward and somatosensory regions [17].

Recent functional neuroimaging studies using AN subjects suggested impairment in reward system and/or cognitive control brain areas, which are salient phenotypic characteristics of the disorder. In terms of the reward system, AN patients show increased dopamine receptor functioning during recovery [18], but demonstrate dopamine dysfunction as reflected in dopaminergic hypoactivity within striatal regions when processing pleasant stimuli [4]. This would suggest that one underlying mechanism for AN is related to anhedonia, reflected in impairment of experiencing reward or pleasure. However, this conclusion is not always consistent with other recent studies showing that AN patients show increased reward-related activity in response to images of underweight women [19] and increased activity in other reward-related regions (i.e., insula and orbitofrontal cortex) for food stimuli [20–21].

Conversely, reduced prefrontal cortex (PFC) activity has often been observed among AN patients compared to healthy controls (HCs) using functional neuroimaging techniques [22–25]. The PFC is a key region for executive functioning, which includes inhibitory control, working memory, and planning. Behavioral studies have revealed that AN patients often have difficulties with tasks requiring inhibitory control, including response inhibition and task switching [26]. Thus, inhibitory control might be a key component influencing the salient characteristics of AN. Functional imbalance between reward and inhibitory control in the cerebral cortex, especially the PFC, might be related to AN susceptibility or pathology. However, regional structural relevance regarding functional abnormalities in AN remain largely unknown [27–28]. Moreover, the PFC is one of the last brain regions to fully develop relative to subcortical regions [29]. PFC organization undergoes dynamic changes in terms of synaptic density throughout prepubescence and adolescence [30]. This might be one key reason why peak AN onset tends to be in the teenage years.

The purpose of the present study was to investigate changes in brain morphology using a VBM approach to address mechanisms underlying the emergence of AN during adolescence. We predicted that AN during adolescence reflects a developmental trajectory among brain regions involved in reward and executive functioning. We investigated differences in GMV using an unbiased, whole-brain, voxel-by-voxel approach. Control subjects were used as a comparison group. Several covariates, including age, age at onset and disease duration, were used to control for other factors that could influence brain development. The current study also assessed whether alterations in regional GMV might be correlated with intellectual ability.

## Methods

### Participants

Participants included 20 un-medicated girls with AN (mean age, 14.2 years; standard deviation [SD], 1.81 years; age range, 12–17 years), who were referred to our laboratory between 2008 and 2011. All patients satisfied diagnostic criteria for the restrictive subtype of AN based on the Diagnostic and Statistical Manual of Mental Disorders, Fourth Edition, Text Revision (DSM-IV-TR #307.1) and had amenorrhea. To exclude other psychiatric conditions, a licensed child and adolescent psychiatrist administered a Structured Clinical Interview for DSM-IV Axis I Disorders (SCID-I) and the Mini-International Neuropsychiatric Interview for Children and Adolescents (MINI-KID) [31, 32]. Mean Body Mass Index (BMI) for the AN group was 14.4; SD, 2.08.

Matched controls comprised 14 healthy girls (mean age, 14.9 years; SD, 1.59 years; age range, 11–16 years), who were recruited from the community (primarily from school samples). Age, handedness, and full scale intelligence quotient (FSIQ) matched between the AN and control groups. None of the participants (in either group) had any psychiatric psychopathology or head trauma. All participants were right-handed and un-medicated. All scans in both groups were carried out on the same machine over the same time-period. An additional exclusion criterion for both healthy and AN subjects was an FSIQ below 75, as measured by the Wechsler Intelligence Scale for Children—Fourth Edition (WISC-IV) [33].

The Committee of Life Ethics, Graduate School of Medicine, Kumamoto University, approved the study protocol. The parents of all participants gave written informed consent. The experimental protocol was conducted in accordance with the Declaration of Helsinki.

### Brain imaging and analysis

High-resolution, sagittal T1-weighted and coronal FLAIR magnetic resonance (MR) imaging datasets were obtained using a Trio scanner (3T; Siemens Medical Solutions, Siemens AG, Erlangen, Germany). An inversion-prepared three-dimensional multi-planar rapidly acquired gradient echo (MPRAGE) sequence and an eight-element phased-array RF reception coil (Siemens AG) was also employed.

A generalized autocalibrating partially parallel acquisition (GRAPPA) and processing were used to reduce scan time, with a GRAPPA factor of 2. Scan parameters were as follows: echo time, 2.74 ms; repetition time, 2.1 s; inversion time, 1.1 s; flip angle, 12°; three-dimensional matrix, 256 × 256 × 128; field of view, 256 × 256 × 170 mm; bandwidth, 48.6 kHz; scan time, 4 min 56 s. Licensed neuroradiologists conducted assessment of the FLAIR or T1-weighed images, completing their evaluations before the neuroimaging study began.

Voxel-based morphometry (VBM) is a fully automated whole-brain morphometric technique used to detect regional structural differences between groups on a voxel-by-voxel basis [34–35]. The VBM was performed using Statistical Parametric Mapping version 8 (SPM8) software (Wellcome Department of Imaging Neuroscience, University College London, London, UK; http://www.fil.ion.ucl.ac.uk/spm/software/spm8/) implemented in MATLAB 9.5 (MathWorks Inc., Natick, MA). Images were segmented coarsely into gray matter, white matter, cerebrospinal fluid, and skull/scalp compartments using tissue probability maps. A standard template was conformed to the space defined by the International Consortium for Brain Mapping [36–37], National Institutes of Health P-20 project, and approximates the space described in the Talairach and Tournoux atlas [38]. The transform for this normalization was used to rewrite the original image into the standard space. Volume changes induced by normalization were adjusted via a modulation algorithm. Spatially normalized images were segmented into gray and white matter and then smoothed using a 12-mm full-width half-maximum isotropic Gaussian kernel. Regional GMV differences between groups were analyzed using a general linear model. Potential confounding effects of age and whole segment GMV were modeled, and attributable variances were excluded [39]. The resulting set of voxel values was used to generate a statistical parametric map of *t*-statistics (SPM{t}), which was transformed to a unit normal distribution (SPM{Z}) for comparison. The statistical threshold was set at *p* < .05, with correction for multiple comparisons at the cluster level (height threshold of *Z* > 3.09), given an increased sensitivity within clusters to detect spatially extended signal changes [40–41]. Inference testing was based on Gaussian field theory [42]. Potential problems related to non-isotropic smoothness, which can invalidate cluster-level comparisons [34], were corrected by adjusting the cluster size from the resel per voxel image [40, 43].

### Statistical analyses

SPM8 was used to identify regions that differed significantly between groups and to assess the association between alterations in GMV in an identified cluster. Clinical values were compared in both groups using two-tailed *t*-tests. Data are expressed in terms means ± SD. All statistical tests (ANOVA or ANCOVA) were two-sided; *p* values less than .05 were inferred as statistically significant. Statistical analyses were performed using IBM SPSS 20.0 for Windows (Statistical Package for the Social Sciences; IBM). Exploratory correlation analyses were used to assess whether regional differences in GMV could account for a significant portion of observed variance. Similarly, additional correlation analyses were performed to identify possible associations between GMV and demographic variables within the AN group.

## Results

### Subject characteristics

Characteristics related to the AN and control participants are summarized in Table 1. The AN and control groups were well matched in terms of age (*t* = 1.29, *p* = .21) and FSIQ (*t* = 1.08, *p* = .29). AN participants had a lower average BMI; BMI was not assessed in the control group. The mean disease duration was 12.6 months (SD, 1.82; range, 5–69). None of our patients exhibited evidence of focal structural abnormalities on FLAIR or T1-weighed MRI scans.

**Table 1:** Clinical variables assessed among anorexia and control participants.

	Anorexia (n = 20)		Controls (n = 14)				
	Mean	S.D.	Mean	S.D.	df	t-score	p-value
Age (years)	14.15	1.814	14.93	1.592	32	1.29	0.21
FSIQ	96.10	12.60	100.07	6.855	32	1.08	0.29
BMI	14.35	2.084					
Age at onset (years)	12.60	1.818					
Duration (months)	23.55	17.022					

Abbreviations: S.D., standard deviation; df, degree of freedom; FSIQ, full scale intelligence quotient; BMI, body mass index;

### Global volume changes

Two-tailed independent *t*-tests assessing group differences in total GMV (tGMV) revealed significant effects (Table 2). The AN group showed a significant, 10.0% decrease in tGMV as compared to the control group.

**Table 2:** Volumetric variable comparisons between the anorexia and control groups.

Volumes (ml)	Anorexia (n = 20)		Controls (n = 14)		Mean	ANCOVA	
	Mean	S.D.	Mean	S.D.	difference	Statistics	p-value
Total GMV^a^	686.08	66.202	762.11	35.020	-10.0%	t(32) = 3.92	< 0.001
Left IFG (BA45/46)^b^	0.88	0.092	1.09	0.059	-19.1%	F(1,31) = 12.82	< 0.001
Right IFG (BA45/46)^b^	0.45	0.050	0.55	0.037	-17.6%	F(1,31) = 5.12	< 0.05

^a^ Student's paired t-test was used to compare total GMV between groups.

^b^ ANCOVA was used to compare local GMV between groups while controlling for total GMV as covariate.

Abbreviations: GMV, gray matter volume; IFG, inferior frontal gyrus; BA, Brodmann area; S.D., standard deviation; ANCOVA, analysis of covariance.

### Region-specific GM changes

**Fig 1:**
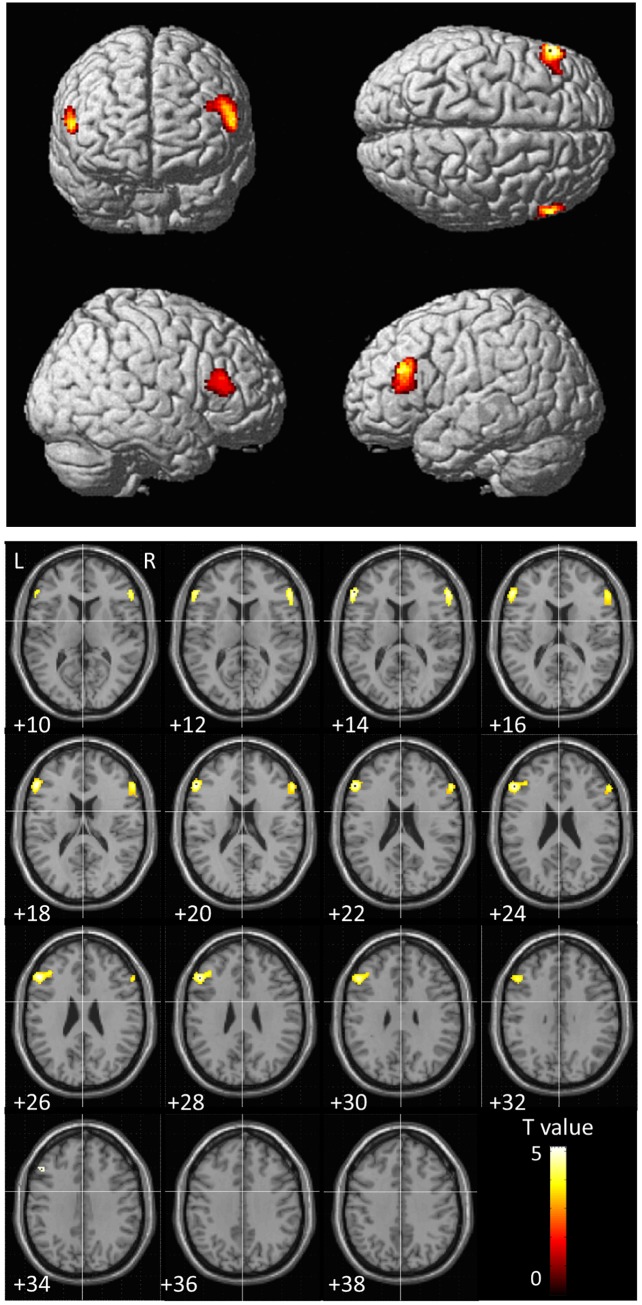
Voxel based morphometry results. Locations displaying significant differences between AN and control participants in regional GMV. Significantly lower GM densities among AN participants were revealed in the bilateral IFG (Brodmann area 45/46). Color scale: 0–5 represents t-values.

After correcting for age and tGMV, regional GMV decreases in the bilateral inferior frontal gyrus (IFG) were observed among AN participants (Table 2). An analysis of region-specific GM changes (ANCOVA with age and total GMV as control variables) yielded two clusters in the bilateral IFG (Fig 1; Table 3). The most prominent neural finding was a significant decrease in GMV in the left IFG in patients with AN (Brodmann area 45/46 [BA45/46]; Talairach’s coordinates x = -52, y = 30, z = 24, Z = 4.40, k = 434 voxels; *p* < .001 for height, FWE corrected to *p* < .05 for multiple comparisons). The mean decrease in GMV in this area was 19.1% among AN patients. Similarly, significant decreases in GMV in the right IFG were observed in the AN group (BA45/46; Talairach’s coordinates x = 58, y = 30, z = 20, *Z* = 4.04, cluster size = 225 voxels; *p* < .001 for height, corrected to *p* < .05 for multiple comparisons using cluster size). The mean decrease in GMV in this area was 17.6%. No other area showing decreased GMV was found to have a corrected cluster probability value approaching significance.

**Table 3:** Regional gray matter volume decreases in AN patients compared with the control group.

		MNI coordinates				Cluster size
Region (Brodmann area)	Side	x	y	z	Z-score	kE (voxels)
Inferior frontal gyrus (45/46)	L	-52	30	24	4.40	434
Inferior frontal gyrus (45/46)	R	58	30	20	4.04	225

### Correlations between GM changes and clinical variables among AN patients

Correlation analyses within the AN group were conducted to assess the relationship between bilateral IFGs (BA 45/46) and clinical variables (age, FSIQ, BMI at the time of scanning, age at onset, duration of AN, and tGMV). As presented in Table 4, significant correlations were found between regional GMVs in the bilateral IFG and age, BMI, age at onset, and tGMV. To exclude the effects of tGMV and BMI related to GMVs in the IFG and clinical variables, partial correlations were analyzed. After correcting for tGMV and BMI, there was a moderate negative correlation between the GMV of each IFG and age (left: *r* = -.475, *p* < .05; right: *r* = -.601, *p* < .01) and age at onset (left: *r* = -.678, *p* < .01; right: *r* = -.570, *p* < .05) (Table 4). Meanwhile, significant correlations between the GMV of each IFG and age were not found in the HC group (left: *r* = .169, *p* > .10; right: *r* = .102, *p* > .10).

**Table 4:** Correlations between volumetric and clinical variables within the AN group.

		Left IFG (BA45/46)	Right IFG (BA45/46)
Correlation	Age	**-0.481** *	**-0.631** **
	FSIQ	-0.015	-0.098
	BMI	**0.564** *	**0.382** #
	Age at onset	**-0.473** *	**-0.473** *
	Duration	0.032	-0.123
	Total GMV	**0.817** ***	**0.881** ***
Partial correlation^a^	Age	**-0.475** *	**-0.601** **
	Age at onset	**-0.678** **	**-0.570** *

^#^ p < .10,

* p < .05,

** p < .01,

*** p < .001

^a^ BMI and total gray matter volume as covariates.

Abbreviations: IFG, inferior frontal gyrus; BA, Brodmann area; FSIQ, full scale intelligence quotient; BMI, body mass index; GMV, gray matter volume.

## Discussion

The aim of the present study was to examine global and local structural GM alterations in patients with AN during adolescence using VBM. Results revealed a significant decrease in total GMV among the AN group compared with the control group. Moreover, after controlling for age and tGMV, regional GMV decreases in the bilateral IFG were observed among the AN group. This unexpected finding emerged from a global VBM analytical approach. In addition, the volume decrease in IFG had a negative association with age and disease onset; this association was not found among the control group. These results suggest that volumetric decreases in the IFG might be involved in the impaired cognitive control of impulsivity observed in patients with AN. Furthermore, volumetric decreases in the IFG are likely dependent on age, suggesting that AN symptom progression may have a significant developmental component.

Several studies have indicated individuals suffering AN also exhibit pathological impulsivity; however, this is more common among individuals suffering bulimia nervosa. Askenazy et al. (1998) found that AN subjects show high rates of impulsive behavior (e.g. non-premeditated suicide attempts, self-harm, kleptomania, and alcohol use) and significantly higher impulsivity scores compared to control group subjects [44]. In the current study, we found that the AN group exhibited a reduced bilateral IFG volume, which was significantly involved in several executive functions (including response inhibition and cognitive control). Given previous studies demonstrating that the right IFG is highly involved in the inhibitory control of motor responses [45–46], recent reviews suggest that the region is specifically involved in the inhibitory control of inappropriate behaviors [47]. Similarly, a previous VBM study of adult AN subjects has reported regional GMV decreases in the right IFG [48]. Although there is less reported evidence of a role for the left IFG in inhibitory behavioral control, recent brain imaging studies have shown that the left inferior frontal region is involved in impulsivity regulation in healthy adults [49], delayed recruitment of inhibitory control in healthy adolescents [50], and deficits of cognitive control in problem gamers [51]. To the best of our knowledge, the present study is the first to suggest volumetric decreases in either the left or right IFGs in adolescents with AN. Our findings suggest that decreases in these regions influence AN pathogenesis and/or severity.

Previous functional imaging studies have reported reduced PFC activity in adult AN subjects when engaged in a cognitive control task, relative to HCs [22–25]. While those studies have shown that AN patients need less inhibitory control to maintain behavioral performance, a recent resting-state fMRI study of AN patients demonstrated functional alterations in the bilateral IFG [52]. Specifically, this study demonstrated functional connectivity from the right IFG to the midcingulum and from the bilateral orbitofrontal gyrus to the right IFG in patients with AN. Additionally, altered functional connectivity from the bilateral insula to the left IFG was observed, indicating a specific connection between the cognitive control system and regions involved in basic motivational processing [52].

The present study also assessed correlations between GM volume changes and clinical variables within the AN group, revealing significant associations between regional GMV in the bilateral IFG and FSIQ, age, age at onset, BMI, and tGMV. Moreover, after controlling for tGMV and BMI, there was a moderate negative correlation between IFG volume and both age and age at onset. These results indicate that particular psychophysiological factor(s) during adolescent development might be closely tied to AN progression and severity. For adult eating disorder patients, most functional imaging studies have reported the IFG as a region demonstrating high neural activity. We were unable to fully clarify specific underlying mechanisms related to GMV and AN emergence in adolescence. However, it is well known that brain maturation during adolescence is characterized by elimination and refinement of synaptic connections in the cerebral cortex, resulting in decreased GM density [53]. Therefore, we speculate that precocious or unbalanced refinement of neuronal connections within the IFG might be an important factor related to pathogenesis of AN during adolescence.

VBM studies provide an unbiased assessment of regional alterations in GMV. However, these studies have a significant number of limitations that must be acknowledged and controlled for as much as possible. Care was taken to make sure that there were no issues with alignment. Subjects in the two groups had an almost identical mean age, and were selected from a narrow age range to minimize any potential developmental differences in template registration. It should be noted that BMI may influence GMV measurements, as it was recently reported that structural brain differences between AN and HC may simply reflect a substantial difference in body weights [54]. Unfortunately, BMI was not assessed in the control group. However, several previous studies have reported an association between BMI and total GMV in AN, suggesting that total GMV likely reflects the patient’s BMI in a reciprocal manner [14, 55]. Moreover, recent studies assessed brain imaging differences between AN and HC groups using total GMV as a confounding factor instead of BMI [56, 57]. Therefore in this study, we likewise used total GMV, as well as age, as confounding factors for VBM analysis. Therefore, the potentially confounding influence of differences in BMI between AN patients and HCs seems to have least been partly accounted for in the present study.

A second notable limitation of the present study was the small sample size of the HC group. This is perhaps why we did not observe any significant association between age and volumetric IFG reduction within the control sample. Although we speculate that this age-related reduction is observed among healthy adolescent females, the reduction slope with aging is much smaller when compared to decreases observed among AN patients.

Finally, we did not obtain an eating disorder inventory for the AN group. Therefore, a possible association between the severity of AN and the regional volume reductions observed in our study could not be investigated. Additional studies that carefully account for multiple clinical assessments will be required to demonstrate such a relationship.

In conclusion, the present study found significant tGMV and regional GMV decreases in the bilateral IFG in adolescent individuals with AN. This GM decrease, which is inferred to be within a region highly relevant to inhibitory control, might explain the presence of impulsive behaviors observed in AN. Further research is necessary to confirm these findings and clarify the causes and consequences of such structural alterations.
